# ENO1 as a potential tumor prognostic marker promotes the proliferation, invasion, and metastasis of breast cancer cells

**DOI:** 10.1016/j.gendis.2026.102219

**Published:** 2026-04-28

**Authors:** Lifang He, Yue Xu, Lu Huang, Lijuan He, Zexiao Chen, Zhaochang Qi, Yukun Cui

**Affiliations:** a Breast Center, Cancer Hospital of Shantou University Medical College, Shantou, Guangdong 515063, China; b Shantou Key Laboratory of Precision Diagnosis and Treatment in Women's Cancer, Cancer Hospital of Shantou University Medical College, Shantou, Guangdong 515063, China; c State Key Laboratory of Respiratory Disease, Guangzhou Medical University, Guangzhou, Guangdong 511436, China; d Guangzhou Morui Pharmaceutical Technology Co., Ltd., Guangzhou Medical University, Guangzhou, Guangdong 511436, China; e Precision Oncology Diagnosis and Treatment Centre of Foshan Fosun Chancheng Hospital, Foshan, Guangdong 528031, China; f Outpatient Nursing, Hangzhou West Lake Zhijiang Ophthalmology Hospital, Hangzhou, Zhejiang 310000, China

Breast cancer (BRCA) is the leading cause of cancer-related death in women.^1^ Recent studies have shown that the metabolism of BRCA is related to its growth, proliferation, invasion, chemoresistance, and poor response to treatment.^2^ Aerobic glycolysis (also termed the Warburg effect) is an important type of cancer metabolism.^3^ Enolase 1 (ENO1) is a glycolytic enzyme catalyzing the conversion of 2-phosphoglycerate to phosphoenolpyruvate. Overexpression of ENO1 activates glycolysis and triggers the development of cancers.^4^ However, the clinical significance and function of ENO1 in BRCA remain unclear. In this study, we aimed to investigate the clinical relevance of ENO1 in BRCA and to determine its role in the proliferation, invasion, and metastasis of BRCA cells.

To explore the clinical relevance of ENO1 in BRCA, we first analyzed the expression of ENO1 based on The Cancer Genome Atlas database. ENO1 expression was significantly elevated in BRCA samples versus the non-adjacent and adjacent normal tissues (Fig. 1A and B). Kaplan–Meier plotter results revealed that high ENO1 expression was correlated with poorer prognosis in BRCA (Fig. 1C; Fig. S1A and S1B). UALCAN database also revealed that higher ENO1 mRNA levels predicted worse overall survival in BRCA (Fig. S1C).

**Figure 1:**
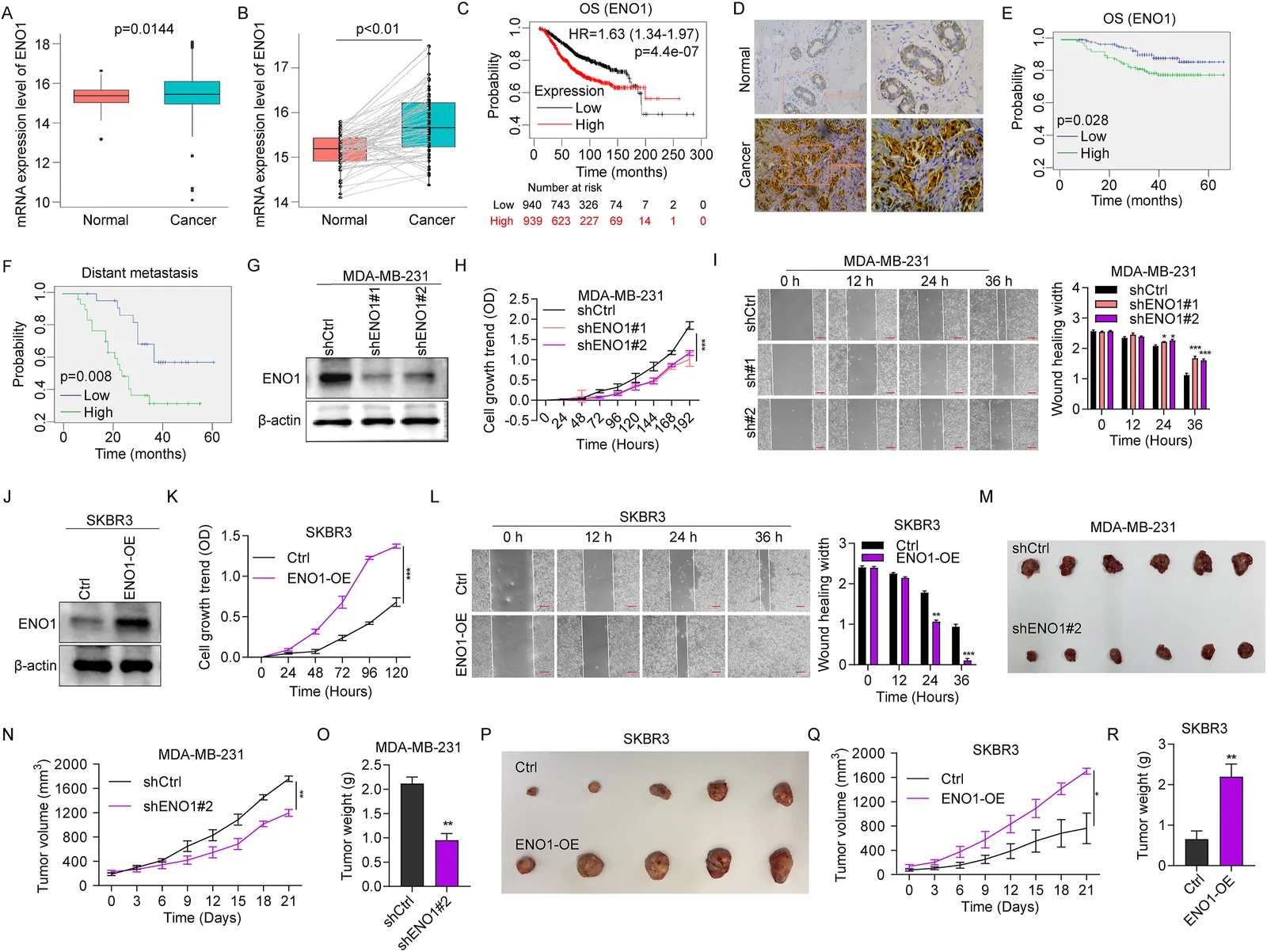
ENO1 promotes the proliferation, migration, and tumor progression of breast cancer (BRCA). **(A, B)** TCGA database analyses of ENO1 expression in unpaired and paired BRCA tissues and adjacent normal tissues. **(C)** Overall survival curves using the Kaplan–Meier plotter. **(D)** Immunohistochemistry staining of ENO1 in BRCA tissues and adjacent normal tissues. Representative images are shown. Scale bar: 50 μm. **(E, F)** Kaplan–Meier overall survival curves based on (E) 293 patients and (F) 55 patients with distant metastasis were drawn using the SPSS tool. **(G)** Western blotting assays showing ENO1 expression after ENO1 knockdown in MDA-MB-231 cells. **(H)** CCK-8 assays showing the effect of ENO1 knockdown on the proliferation of MDA-MB-231 cells. **(I)** Wound healing assays showing the cell migration ability in MDA-MB-231 cells *in vitro*. **(J)** Western blotting assays showing ENO1 expression after overexpressing ENO1 in SKBR3 cells. **(K)** CCK-8 assays showing the effect of ENO1 overexpression on the proliferation of SKBR3 cells. **(L)** Wound healing assays showing the cell migration ability in SKBR3 cells *in vitro*. **(M)** MDA-MB-231 xenograft tumor at the end of the experiment. **(N, O)***In vivo* tumor growth curves and tumor weight for each group in MDA-MB-231 xenograft models. **(P)** SKBR3 xenograft tumor at the end of the experiment. **(Q, R)***In vivo* tumor growth curves and tumor weight results for each group in SKBR3 xenograft models. ∗*p* < 0.05, ∗∗*p* < 0.01, and ∗∗∗*p* < 0.001.

To investigate the clinical significance of ENO1 in Chinese BRCA patients, we collected 293 tissue samples from patients with BRCA and subjected them to immunohistochemistry staining of ENO1. The expression of ENO1 was higher in BRCA tissues versus adjacent normal tissues (Fig. 1D). There were no significant correlations between ENO1 expression and clinicopathological features, including age, menopausal status, endocrine treatment status, endoplasmic reticulum status, and progesterone receptor status (Table S1). Higher ENO1 levels predicted worse overall survival in BRCA (Fig. 1E). Additionally, we explored the relationship between ENO1 expression and tamoxifen or other endocrine therapy status. There was no significant association between ENO1 levels and prognosis in patients with BRCA receiving tamoxifen and other endocrine therapy (Fig. S2A and S2B). However, high ENO1 expression was significantly associated with poor prognosis in patients who did not receive endocrine therapy (Fig. S2C). Moreover, high ENO1 expression was correlated with poor prognosis in patients with distant metastasis (more than two lymph node metastases), but not in those without distant metastasis (Fig. 1F; Fig. S2D–S2F). Among patients with late-stage BRCA (stages III–IV), those with high ENO1 expression had a poor prognosis (Fig. S2G and S2H).

Next, we investigated the role of ENO1 and tamoxifen resistance in BRCA. ENO1 protein levels were higher in MCF-7-TR cells compared with MCF-7 cells (Fig. S3A). We overexpressed ENO1 in MCF-7 cells and knocked down ENO1 in MCF-7-TR cells (Fig. S3B–S3E). The sensitivity of MCF-7-TR-ENO1-shRNA or MCF-7-ENO1-OE cells to tamoxifen was not changed compared with that of control cells (Fig. S3F–S3K). Collectively, ENO1 expression was not related to tamoxifen resistance in BRCA cells.

We then explored the role of ENO1 in the development of BRCA. The expression of ENO1 was higher in MDA-MB-231 cells (Fig. S4A). Subsequently, we constructed ENO1 stable knockdown MDA-MB-231 cell lines (Fig. 1G). The proliferative, migration, and invasion ability of MDA-MB-231 was suppressed by ENO1 down-regulation (Fig. 1H and I; Fig. S4C). By contrast, overexpression of ENO1 contributed to the enhanced proliferation, migration, and invasion of SKBR3 cells (Fig. 1J–L; Fig. S4B). Moreover, we observed that the expression of epithelial–mesenchymal transition (EMT) pathway-related proteins, including β-catenin, Vimentin, Snail, and Twist, was positively regulated by ENO1, while the expression of E-cadherin was negatively regulated by ENO1, in BRCA cells (Fig. S4D and S4E). Mechanistically, ENO1 overexpression enhanced glycolytic activity in BRCA cells, accompanied by increased lactate production and elevated histone H3 lysine 18 lactylation (Fig. S5A–S5F). Pharmacological inhibition of glycolysis markedly attenuated ENO1-induced histone lactylation, EMT-associated transcriptional programs, and invasive capacity, supporting a glycolysis-dependent contribution of ENO1 to EMT-associated phenotypes, at least in part in association with histone lactylation (Fig. S5G–S5L).

Finally, animal experiments were used to explore the effect of ENO1 on tumor development *in vivo*. Compared with the control group, the tumor proliferation rate of the ENO1-knockdown group was significantly reduced (Fig. 1M). The volume and mass of tumors in the ENO1-knockdown group were markedly lower than those recorded in the corresponding control group (Fig. 1N and O). Compared with the control group, the ENO1-overexpression group had elevated tumor proliferation rate, tumor volume, and tumor mass (Fig. 1P–R). In addition, we evaluated the effect of ENO1 on BRCA metastasis in nude mice. We observed that, compared with the control group, the SKBR3-ENO1-OE group accounted for a larger proportion of cancer metastases in the lung and lymph nodes (Fig. S6A and S6C). However, compared with the control group, MDA-MB-231-ENO1-shRNA cells accounted for a significantly smaller proportion of cancer metastases in the lung and lymph nodes (Fig. S6B and S6D). Taken together, these findings indicated that ENO1 overexpression was critical for tumor growth and metastasis *in vivo*.

In conclusion, high ENO1 expression was correlated with the metastasis and poorer survival of BRCA patients. Overexpression of ENO1 supported the proliferation, invasion, and metastasis of BRCA cells *in vitro* and *in vivo*. ENO1 is a promising biomarker and therapeutic target for BRCA patients.

## CRediT authorship contribution statement

**Lifang He:** Writing – original draft, Resources, Project administration, Funding acquisition. **Yue Xu:** Writing – original draft. **Lu Huang:** Resources. **Lijuan He:** Data curation. **Zexiao Chen:** Data curation. **Zhaochang Qi:** Data curation. **Yukun Cui:** Project administration, Funding acquisition, Conceptualization.

## Ethics declaration

This study was approved by the medical ethics committee of the Cancer Hospital of Shantou University Medical College (2023104). The need for informed consent was waived by the medical ethics committee of the Cancer Hospital of Shantou University Medical College. The animal protocol was approved by the animal care and use ethical committee of Cancer Hospital of Shantou University Medical College (SUMC2021-075).

## Data availability

The datasets used and/or analyzed during the current study are available from the corresponding author on reasonable request.

## Funding

This work was supported by the Science and Technology Special Fund of Guangdong Province of China (No. 210728156901648, 210729156901814), the National Natural Science Foundation of China (No. 82272670), and the National Natural Science Foundation Cultivation Project of Cancer Hospital of Shantou University Medical College (Guangdong, China) (No. 2023GP005).

## Conflict of interests

The authors declared no competing interests.
