# Supplementary material for: ENO1 as a potential tumor prognostic marker promotes the proliferation, invasion, and metastasis of breast cancer cells
Source: Genes Dis. 2026 Apr 28;14(1):102219. doi: 10.1016/j.gendis.2026.102219 (PMC13571238; doi:10.1016/j.gendis.2026.102219)
Supplement: Supplementary file 1 [file mmc1.docx]

**Materials and methods**

**Immunohistochemistry (IHC)**

For IHC, complete clinicopathological features and follow-up data were retrieved for 293 patients with breast cancer from May 2009 to July 2012 at the Cancer Hospital Affiliated to Medical College of Shantou University. The clinical tissues of patients with breast cancer were fixed, embedded, and cut into sections (thickness: 4 μm). After soaking in xylene for dewaxing and hydration, the sections were immersed in sodium citrate buffer for antigen retrieval. Next, the sections were blocked with goat serum (catalog number [cat#]: AR0009; Boster Biological Technology) and incubated overnight with primary antibody (cat#: CC36131; Bioworld Technology). After washing the primary antibody, the sections were incubated the secondary antibody (cat#: GK500510A; Gene Tech Shanghai Company Limited, Shanghai, China) at room temperature for 30 min. After washing the secondary antibody, DAB was added (cat#: DAB-0031; Maixin Biotechnology) to develop the color of tissue sections, and hematoxylin dye (cat#: H9627; Merck,) was used to stain the nucleus. After adding neutral gum (cat#: HC0782; Biofount,) to seal the slice, the staining intensity was observed according to the former report ^1^.

**Cell culture**

The MCF-7, MDA-MB-231, SKBR3 cell lines were purchased from the Culture Collection of the Chinese Academy of Sciences, Shanghai, Chia. TAM-resistant MCF-7 (MCF-7-TR) cell line was obtained from Guangdong Provincial Key Laboratory of Breast Cancer Diagnosis and Treatment (Guangzhou, China). The induction method involved a continuous increase in the dose of 4-OHT from 10 nM to 8 μM in phenol red-free Dulbecco’s modified Eagle’s medium (DMEM; cat#: C11995500BT; Gibco, China) for 6 months. Once a significant population of cells were enriched to grow in the presence of 2, 4, and 8 μM of 4-OHT, the cells were adaptively cultured to grow in 2, 4, and 8 μM of 4-OHT containing phenol red-free DMEM for another 3 months to stabilize the phenotypes ^2^. MCF-7 cells were cultured in DMEM supplemented with 10% fetal bovine serum (FBS). The other cells were cultured in DMEM supplemented with 10% FBS. All cells were maintained at 37℃ in a humidified atmosphere containing 5% CO_2_.

**Transfection**

Three different guiding RNA pairs for ENO1 knockdown and non-targeting controls, the plasmids for ENO1 overexpression and control were purchased from Beijing Hesheng Gene Technology (Beijing, China). Stable cell lines were established by infecting MDA-MB-231 and SKBR3 cells with lentivirus, followed by puromycin selection.

**Quantitative reverse transcription-polymerase chain reaction**

According to the instructions, total RNA was obtained from cultured cell lines by lysis with TRIzol reagent (cat#: 15596026; Invitrogen,). cDNA was synthesized using the TaKaRa PrimeScript^TM^ RT Reagent Kit with gDNA Eraser (cat#: RR047A; Takara Bio). Primers used for amplification of ENO1 and β-actin were as follows: ENO1, forward, 5’-GAGATCTCGCCGGCTTTAC-3’, reverse, 5’- TCAACAGCCTTTGAGACACCCTT-3’; β-actin, forward, 5’-CATGTACGTTGCTATCCAGGC-3’, reverse, 5’-CTCCTTAATGTCACGCACGAT-3’. Relative gene expression was calculated using the 2^−ΔΔCT^ method.

**Western blotting**

Total protein was collected using radioimmunoprecipitation assay (RIPA) lysis buffer (cat#: 0020; Solarbio,). Extracts were centrifuged at 12,000 rpm, at 4℃ for 30 min. Total protein was quantified using the bicinchoninic acid (BCA) Protein Assay Kit (cat#: PC0020; Solarbio). Protein extracts were denatured in 4× Loading Buffer (cat#: P1015; Solarbio) at 98℃ for 10 min. Proteins were separated in 10% sodium dodecyl sulfate-polyacrylamide gel electrophoresis gels (cat#: P1200; Solarbio), and transferred onto 0.45-μm polyvinylidene difluoride membranes (cat#: IPVH00010; Ireland,) for 2 h at 100 V. Membranes were blocked with 5% bovine serum albumin (cat#: A8020; Solarbio) for 1 h and incubated with primary antibodies overnight at 4℃. The primary antibodies were ENO1 polyclonal antibody (cat#: CC36131; Bioworld Technology) and glyceraldehyde-3-phosphate dehydrogenase (GAPDH; cat#: Cst.5174s; Cell Signaling Technology). Subsequently, the membrane was incubated with a goat anti-rabbit horseradish peroxidase-conjugated secondary antibody (1:20,000, cat#: ab97051; Abcam,). Immunoreactive bands were acquired using an ECL Western Blotting Kit (cat#: 34580; Thermo Fisher) and ChemiDoc XRS+ Image Analysis System (Bio-Rad, Shanghai, China).

**Cell Counting Kit-8 (CCK-8) cell proliferation assays**

The breast cancer cell lines were plated on 96-well plates at a density of 1,000 cells per well. The CCK-8 solution (cat#: C0039; Beyotime) was added to each well at the indicated time. After 2.5 h of incubation at 37℃ and 5% CO_2_, absorbance was detected (optical density value) at a wavelength of 450 nm (Thermo MK3; Thermo Fisher). All optical density values were analyzed using ImageJ software (National Institutes of Health, Bethesda, MD, USA).

**Transwell cell invasion assays**

Serum-starved MDA-MB-231-ENO1-shRNA, SKBR3-ENO1-OE, and their controls were seeded in the upper chamber of a Transwell chamber containing 400 μl of serum-free media. Thereafter, they were separated from the chemoattractant (10% FBS-supplemented media) using a polycarbonate membrane (pore size: 8 μm) (cat#: 353097; Corning Inc., Corning NY, USA). Matrigel (cat#: 356234; Corning Inc.) was diluted in serum-free medium (1:8 ratio) and was used to cover the upper chambers. After 24 h, cells in the chambers were fixed in 4% paraformaldehyde, stained with 0.1% crystal violet and counted in quadruplicate fields of view under ×100 magnification.

**Wound healing assays**

SKBR3-ENO1-OE, MDA-MB-231-ENO1-shRNA, and their controls were seeded in six-well plates at a density of 5×10^5^ cells per well. The cells were scratched with a 200-μl plastic pipette tip to create a uniform wound. Thereafter, the wound width was examined following 24 h of incubation under a microscope (Nikon, Tokyo Japan). The cell migration ability was expressed by the closure of the gap distance. The distance was measured using ImageJ software.

**Animal studies**

SKBR3-ENO1-OE, MDA-MB-231-shRNA, and their control cells were collected and suspended in 0.9% NaCl on ice. Female BALB/c nude mice (n=24) were randomly divided into four groups. Cells (1×10^7^) were subcutaneously injected in the armpit of the right forelimb of each nude mice. After the masses in the armpit of nude mice became palpable or visible to the naked eye, tumor volumes were measured once every 3 days. In addition, another 40 BALB/c nude mice aged 4–5 weeks were randomly divided into eight groups (n=5 per group). The above four cell lines were inoculated into the foot pad (1×10^7^ cells) and tail vein (1×10^6^ cells) of the nude mice. Each cell line was inoculated into five nude mice. After 6–8 weeks, the nude mice were sacrificed by cervical dislocation. At end, the tumors were obtained and images were captured. Metastasized lymph node and lung tissue samples were obtained for further hematoxylin-and-eosin (HE) staining experiments. The animal protocol was approved by the animal care and use ethical committee of Cancer Hospital of Shantou University Medical College (SUMC2021-075).

**HE staining**

Subcutaneous tumor and lymph node samples were fixed and embedded in paraffin. The tissues were cut into section (thickness: 4 μm), soaked in xylene for dewaxing, and hydrated. The slices were placed into hematoxylin dye solution for 3–5 min, and washed with tap water before differentiation. Finally, the slices were dehydrated in gradient alcohol, dyed in eosin staining solution for 5 min, and subsequently dehydrated and sealed.

**Glucose uptake and lactate production assay**

Glucose uptake was measured using a 2-NBDG Glucose Uptake Assay Kit (APExBIO, Houston, TX, USA; Cat. No. K2212) according to the manufacturer’s instructions with minor modifications. Briefly, breast cancer cells were seeded into 6-well plates and cultured to 70-90% confluence. Cells were washed twice with phosphate-buffered saline (PBS) and incubated in glucose-free medium for 40 min at 37 °C. After the indicated treatments, cells were incubated with 2-NBDG working solution (prepared by diluting 2-NBDG 1:100 in glucose-free medium) at 37 °C for 20 min in the dark. Cells were then washed with 1× assay buffer and harvested for analysis.Glucose uptake was quantified by flow cytometry using the FITC channel, and data were analyzed using FlowJo software. Relative glucose uptake was calculated based on the mean fluorescence intensity of 2-NBDG.

Lactate production was measured using a lactate assay kit (Abbkine, Wuhan, China; CheKine™ Lactate Assay Kit, Cat. No. KTB1100) according to the manufacturer’s instructions. Briefly, breast cancer cells were cultured in fresh phenol red-free medium under the indicated experimental conditions. Culture supernatants were collected and centrifuged at 800 × g for 5 min to remove cellular debris. For lactate measurement, samples were diluted with lactate assay buffer to a final volume of 50 μL per well in a 96-well plate. Subsequently, 50 μL of working reaction mixture was added to each well, and the plate was incubated at 37 °C for 30 min in the dark. Lactate levels were determined by measuring absorbance at 450 nm using a microplate reader. Lactate concentrations were calculated based on a standard curve generated using the provided L-lactate standard.

**Digital imaging**

Transwell cell invasion images, IHC sections, and HE slides were scanned and collected using the LAS V3.6 image acquisition system of Leica forward fluorescent microscope (DMI3000B; Leica, Japan). The wound healing assay images were collected using a Nikon inverted microscope (Nikon).

**Statistical analysis**

The GraphPad Prism 9.0 software (GraphPad Software Inc., San Diego, CA, USA) was used to analyze data. Two-tailed Student’s *t*-test, repeated measures mixed analysis, or one-way analysis of variance were used as appropriate. A non-parametric test, such as two-tailed Mann–Whitney or Kruskal–Wallis test, was used for samples that did not comply with normality assumption. P-values <0.05 indicate statistically significant differences, and test assumptions were checked for all analyses.

**Table S1:** Relationship of ENO1 expression with clinicopathologic features and biomarkers. 294 patients with breast cancer were included.

| Clinicopathological | ENO1 expression | | Chisq P | | |
| --- | --- | --- | --- | --- | --- |
| features | Low(＜=3)n(%) High(＞3)n(%) | |  |  |  |
| Age (years) |  |  |  |  |  |
| <50 | 64(46.0) | 75(54.0) | 2.448 | 0.118 |  |
| >=50 | 85(55.2) | 69(44.8) |  |  |  |
| Menopausal status |  |  |  |  |  |
| Pre- | 65(46.1) | 76(53.9) | 2.294 | 0.13 |  |
| Post- | 83(55.0) | 68(45.0) |  |  |  |
| Endocrine treatment |  |  |  |  |  |
| TAM | 42(48.3) | 45(51.7) | 1.077 | 0.584 |  |
| Other | 43(55.8) | 34(44.2) |  |  |  |
| Clinical tumor size |  |  |  |  |  |
| ＜=4 | 104(49.5) | 106(50.5) | 0.538 | 0.463 |  |
| ＞4 | 44(54.3) | 37(45.7) |  |  |  |
| Histological grade |  |  |  |  |  |
| I | 10(40.0) | 15(60.0) | 3.313 | 0.191 |  |
| II | 34(43.0) | 45(57.0) |  |  |  |
| III | 74(54.0) | 63(46.0) |  |  |  |
| Histological type |  |  |  |  |  |
| Ductal | 132(49.4) | 135(50.6) | 2.871 | 0.238 |  |
| Lobular | 9(69.2) | 4(30.8) |  |  |  |
| Other | 6(66.7) | 3(33.3) |  |  |  |
| Tumor stage |  |  |  |  |  |
| T0-T2 | 108(49.3) | 111(50.7) | 0.379 | 0.538 |  |
| T3-T4 | 38(53.5) | 33(46.5) |  |  |  |
| Regional lymph nodes |  |  |  |  |  |
| Yes | 76(45.5) | 91(54.5) | 3.683 | 0.058 |  |
| No | 70(37.7) | 53(62.3) |  |  |  |
| TNM stage |  |  |  |  |  |
| I-II | 76(52.4) | 69(47.6) | 0.504 | 0.478 |  |
| III-Ⅳ | 67(48.2) | 72(51.8) |  |  |  |
| ER |  |  |  |  |  |
| Negative | 58(46.0) | 68(54.0) | 2.056 | 0.152 |  |
| Positive | 91(54.5) | 76(45.5.0) |  |  |  |
|  | 149(50.9) | 144(49.1) |  |  |  |
| PR |  |  |  |  |  |
| Negative | 87(53.7) | 75(46.3) | 1.046 | 0.306 |  |
| Positive | 61(47.7) | 67(52.3) |  |  |  |
| HER2 |  |  |  |  |  |
| Negative | 96(56.1) | 75(43.9) | 4.663 | 0.031 |  |
| Positive | 51(43.2) | 67(56.8) |  |  |  |
| Ki-67(%) |  |  |  |  |  |
| <=14 | 36(50.7) | 35(49.3) | 0.001 | 0.977 |  |
| >14 | 113(50.9) | 109(49.1) |  |  |  |
| P53 |  |  |  |  |  |
| 1+ | 23(37.1) | 39(62.9) | 3.225 | 0.199 |  |
| 2+ | 22(55.0) | 18(45.0) |  |  |  |
| 3+ | 31(46.3) | 36(53.7) |  |  |  |
| VEGF-C |  |  |  |  |  |
| 1+ | 44(47.3) | 49(52.7) | 0.695 | 0.706 |  |
| 2+ | 36(45.0) | 44(55.0) |  |  |  |
| 3+ | 12(38.7) | 19(61.3) |  |  |  |
| Cancer subtype |  |  |  |  |  |
| Luminal A | 20(48.8) | 21(51.2) | 9.954 | 0.041 |  |
| luminal B1 | 54(58.7) | 38(41.3) |  |  |  |
| luminal B2 | 12(30.0) | 28(70.0) |  |  |  |
| HER-2 | 18(42.9) | 24(57.1) |  |  |  |
| Triple negative | 19(51.4) | 18(48.6) |  |  |  |
| Lymph node number |  |  |  |  |  |
| <=2 | 89(54.6) | 74(45.4) | 2.065 | 0.151 |  |
| >2 | 60(46.2) | 70(53.8) |  |  |  |
| Death |  |  |  |  |  |
| Yes | 15(34.9) | 28(65.1) | 5.142 | 0.023 |  |
| No | 134(53.6) | 116(46.4) |  |  |  |
| Distant metastasis |  |  |  |  |  |
| Yes | 24(43.6) | 31(56.4) | 1.411 | 0.235 |  |
| No | 125(52.5) | 113(47.5) |  |  |  |
| Endocrine treatment |  |  |  |  |  |
| Yes | 85(51.8) | 79(48.2) | 0.141 | 0.707 |  |
| No | 62(49.2) | 64(50.8) |  |  |  |

**Fig S1 ENO1 expression is high in breast cancer, and increased levels predict worse survival.** (**A-B**) Survival curves using the Kaplan–Meier plotter are shown for: (**A**) RFS and (**B**) DMFS. (**C)** Survival curves using the UALCAN database are shown for OS.

**Fig S2** **Kaplan–Meier overall survival curves were drawn according to ENO1 expression using SPSS. (A-C)** Kaplan–Meier overall survival curves based on: **(A)** 87 patients receiving TAM endocrine therapy; **(B)** 77 patients receiving endocrine therapy; and **(C)** 126 patients not receiving any endocrine therapy were drawn using the SPSS tool. (**D-H)** Kaplan–Meier overall survival curves based on: (**D**) 238 patients without distant metastasis; (**E**) 163 patients with less than two metastasizing lymph nodes; (**F**) 130 patients with more than two metastasizing lymph nodes; (**G**) 145 patients with TNM stages I–II; and (**H**) 139 patients with TNM stages III–IV.

**Fig S3** **High ENO1 expression in breast cancer cells is not related to tamoxifen resistance. (A)** Western blot showing ENO1 expression in MCF-7 and MCF-7-TR breast cancer cells. (**B-C)** Western blot and real-time PCR were used to examine the efficiency of ENO1 overexpression in MCF-7 cells. (**D-E)** Western blot and real-time PCR were utilized to examine the efficiency of ENO1 knockdown in MCF-7-TR cells. (**F-H)** CCK-8 assays showing the effect of ENO1 overexpression on cell proliferation in MCF-7 cells cultured with (**F**) 2 μM, (**G**) 4 μM, and (**H**) 8 μM of tamoxifen. **I-K** CCK-8 assays showing the effect of ENO1 knockdown on the proliferation of MCF-7-TR cells within (**I**) 2 μM, (**J**) 4 μM, and (**K**) 8 μM drug resistance. ^**^*p*<0.01, ^***^*p*<0.001.

**Fig S4** **High expression of ENO1 promotes the abilities of breast cancer cells for invasion.** **(A)** Western blot showing ENO1 expression in MDA-MB-231 and SKBR3 breast cancer cells. (**B-C)** Cell invasion ability was evaluated after ENO1 overexpression in (**B**) SKBR3 cells and ENO1 knockdown in (**C**) MDA-MB-231 cells. **(D-E)** Western blot showing the expression of EMT pathway-related proteins after ENO1 knockdown in MDA-MB-231 and ENO1 overexpression in SKBR3 cells. ^***^*p*<0.001.

**Fig S5 ENO1 promotes glycolytic reprogramming and EMT-associated phenotypes in a glycolysis-dependent manner. (A-B)** Glucose uptake was assessed by 2-NBDG staining and flow cytometry in SKBR3 cells with ENO1 overexpression and in MDA-MB-231 cells with ENO1 knockdown. **(C-D)** Lactate production was measured in SKBR3 cells with ENO1 overexpression **(C)** and in MDA-MB-231 cells with ENO1 knockdown **(D)**. **(E–F)** Western blot analysis of histone H3 lysine 18 lactylation (H3K18la) levels in SKBR3 cells with ENO1 overexpression **(E)** and in MDA-MB-231 cells with ENO1 knockdown **(F)**. Total histone H3 was used as a loading control. **(G)** Lactate production was measured in SKBR3 cells with or without ENO1 overexpression following vehicle or 2-DG (5 mM, 24 h) treatment. **(H)** Western blot analysis of H3K18la and total histone H3 levels in SKBR3 cells with ENO1 overexpression treated with vehicle or 2-DG (5 mM, 24 h). **(I–J)** The mRNA levels of EMT-associated transcription factors SNAI1 **(I)** and TWIST1 **(J)** were determined by qRT-PCR in SKBR3 cells with ENO1 overexpression treated with vehicle or 2-DG (5 mM, 24 h). **(K)** Western blot analysis of EMT-associated proteins, including E-cadherin, Vimentin, Snail, and Twist, in SKBR3 cells with ENO1 overexpression treated with vehicle or 2-DG (5 mM, 24 h). **(L)** Cell invasion was assessed by Transwell invasion assay in SKBR3 cells with ENO1 overexpression treated with vehicle or 2-DG (5 mM, 24 h).^**^*p*<0.01, ^***^*p*<0.001.

**Fig S6 High expression of ENO1 promotes the progression of breast cancer.** (**A and C)** HE staining showing the effect of ENO1 overexpression on the (**A**) lung metastatic capacity and (**C**) lymph metastatic capacity of SKBR3 cells *in vivo*. (**B and D)** HE staining showing the effect of ENO1 knockdown on the (**B**) lung metastatic capacity and (**D**) lymph metastatic capacity of MDA-MB-231 cells *in vivo*.

**Reference**

1.	Argote Camacho AX, Gonzalez Ramirez AR, Perez Alonso AJ, et al. Metalloproteinases 1 and 3 as Potential Biomarkers in Breast Cancer Development. *Int J Mol Sci.* 2021;22(16).

2.	Kumari K, Kumar S, Parida DK, Mishra SK. EZH2 knockdown in tamoxifen-resistant MCF-7 cells unravels novel targets for regaining sensitivity towards tamoxifen. *Breast Cancer.* 2021;28(2):355-367.
